# Crystal structure of 22,24,25-trimethyl-8,11,14-trioxa-25-aza­tetra­cyclo­[19.3.1.0^2,7^.0^15,20^]penta­cosa-2,4,6,15(20),16,18-hexaen-23-one

**DOI:** 10.1107/S2056989016020508

**Published:** 2017-01-06

**Authors:** Van Tuyen Nguyen, Hong Hieu Truong, Tuan Anh Le, Anatoly T. Soldatenkov, Tuyet Anh Dang Thi, Thi Thanh Van Tran, Natalia Ya. Esina, Victor N. Khrustalev

**Affiliations:** a Institute of Chemistry, Vietnam Academy of Science and Technology, 18 Hoang Quoc Viet, Hanoi, Vietnam; b Graduate University of Science and Technology, 18 Hoang Quoc Viet, Hanoi, Vietnam; c Department of Biotechnology, Vietnam–Russia Tropical Centre, 58 Nguyen Van Huyen, Hanoi, Vietnam; d Faculty of Chemistry, VNU University of Science, Vietnam National University, 19 Le Thanh Tong, Hoan Kiem, Hanoi, Vietnam; e Organic Chemistry Department, Peoples’ Friendship University of Russia, 6 Miklukho-Maklaya St., Moscow 117198, Russian Federation; f Inorganic Chemistry Department, Peoples’ Friendship University of Russia, 6 Miklukho-Maklay St., Moscow 117198, Russian Federation; g X-Ray Structural Centre, A.N. Nesmeyanov Institute of Organoelement Compounds, Russian Academy of Sciences, 28 Vavilov St., B-334, Moscow 119991, Russian Federation

**Keywords:** crystal structure, macroheterocycles, crown ethers, stacking inter­actions

## Abstract

The title compound, C_24_H_29_NO_4_, is the product of a Petrenko–Kritchenko condensation of 1,5-bis­(2-formyl­phen­oxy)-3-oxa­pentane, pentan-3-one and methyl­ammonium acetate in ethanol. The mol­ecule has mirror symmetry. The aza-14-crown-3 ether ring adopts a *bowl* conformation stabilized by a weak intra­molecular C—H⋯O hydrogen bond. The conformation of the C—O—C—C—O—C—C—O—C polyether chain is t–g^+^–t–t–g^−^–t (t = *trans*, 180°; g = *gauche*, ±60°). The dihedral angle between the benzene rings fused to the aza-14-crown-4-ether moiety is 72.68 (4)°. The piperidinone ring adopts a *chair* conformation. The nitro­gen atom has a trigonal–pyramidal geometry, the sum of the bond angles being 335.9°. In the crystal, the mol­ecules are linked by weak C—H⋯O inter­actions, forming zigzag chains propagating along the [100] direction.

## Abstract 2

Macrocyclic 22,24,25-trimethyl-8,11,14-trioxa-25-aza­tetra­cyclo­[19.3.1.0^2,7^.0^15,20^]penta­cosa-2,4,6,15 (20),16,18-hexaen-23-one obtained by a Petrenko–Kritchenko condensation of 1,5-bis­(2-formyl­phen­oxy)-3-oxa­pentane, pentan-3-one and methyl­ammonium acetate has been studied by X-ray structural analysis.

## Chemical context   

Macroheterocycles containing both crown ether and aza­heterocyclic moieties are prospective compounds not only as metal-ion receptors (Pedersen, 1988 ▸), but also as membrane ion-transporting vehicles (Gökel & Murillo, 1996 ▸), as active components of environmental chemistry (Bradshaw & Izatt, 1997 ▸), for the design of organic sensors (Costero *et al.*, 2005 ▸), in nanosized on-off switches and other mol­ecular electronic devices (Natali & Giordani, 2012 ▸). Moreover, they can possess anti­bacterial (An *et al.*, 1998 ▸) and anti­cancer properties (Artiemenko *et al.*, 2002 ▸; Le *et al.*, 2014 ▸, 2015 ▸), and other useful biological activity (Anh & Soldatenkov, 2016 ▸; Tran *et al.*, 2016 ▸).

Recently, we have developed effective methods for the synthesis of aza­crown ethers containing γ-piperidone (Levov *et al.*, 2006 ▸, 2008 ▸; Anh *et al.*, 2008 ▸, 2012*a* ▸,*b* ▸,*c* ▸; Hieu *et al.* (2011 ▸, 2012*a* ▸,*b* ▸) or γ-aryl­pyridine (Anh & Soldatenkov, 2016 ▸; Tran *et al.*, 2016 ▸) subunits. This chemistry allowed us to make systematic studies of the fine structural features of a novel series of aza­crown macrocycles using X-ray diffraction. Such data should be of use for the subsequent design of more certain drug-like macroheterocyclic mol­ecules bearing new would-be pharmacophore groups.

In attempts to apply this chemistry for obtaining aza­crown ethers which contain a 1,3,5-trimethyl-substituted γ-piperidine moiety, we studied the condensation of di­ethyl­ketone and 1,5-bis­(2-formyl­phen­oxy)-3-oxa­pentane in the presence of methyl­ammonium acetate taken both as the nitro­gen source and as the template in ethanol/acetic acid solution. The reaction proceeded smoothly to give the expected aza­crown title mol­ecule, (I), with 32% yield.
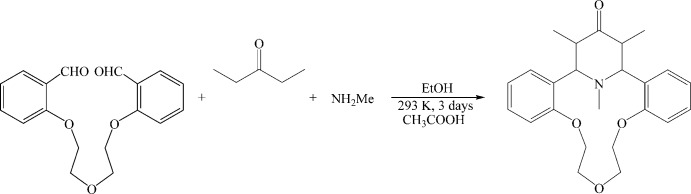


## Structural commentary   

**Figure 1:**
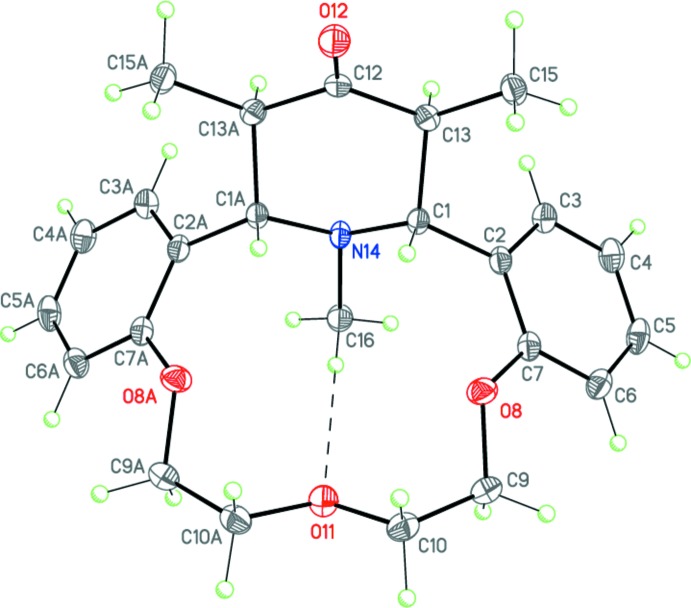
The mol­ecular structure of (I). Displacement ellipsoids are shown at the 50% probability level. H atoms are presented as small spheres of arbitrary radius. The dashed line indicates the intra­molecular C—H⋯O hydrogen bond. Atoms with the suffix *A* are related by the symmetry operation *x*, 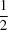 − *y*, *z*.

The title compound (Fig. 1 ▸), the product of a Petrenko–Kritchenko condensation of 1,5-bis­(2-formyl­phen­oxy)-3-oxa­pentane, pentan-3-one and methyl­ammonium acetate in ethanol, crystallizes in the ortho­rhom­bic space group *Pnma* and, in the crystal, occupies a special position on a mirror plane. The aza-14-crown-3 ether ring adopts a *bowl* conformation stabilized by the weak intra­molecular C16—H16*B*⋯O11 hydrogen bond (Table 1 ▸, Fig. 1 ▸). The distances from the center of the macrocycle cavity (defined as the centroid of atoms O8/O11/O8*A*/N14) to the O8, O11 and N14 atoms are 2.265 (2), 1.880 (2) and 2.393 (2) Å, respectively [atoms with the suffix *A* are related by the symmetry operation *x*, 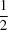 − *y*, *z*.]. The conformation of the C7—O8—C9—C10—O11—C10*A*—C9*A*—O8*A*—C7*A* polyether chain is t–g^+^–t–t–g^−^–t (t = *trans*, 180°; g = *gauche*, ±60°). The dihedral angle between the planes of the benzene rings fused to the aza-14-crown-4-ether moiety is 72.68 (4)°. The piperidinone ring adopts a *chair* conformation. The nitro­gen N14 atom has a trigonal–pyramidal geometry (sum of the bond angles is 335.9°).

The mol­ecule of (I) possesses four asymmetric centers at the C1, C13, C13*A* and C1*A* carbon atoms and can have potentially numerous diastereomers. The crystal of (I) is racemic and consists of enanti­omeric pairs with the following relative configuration of the centers: *rac*-1R*,13S*,13AR*,1AS*.

## Supra­molecular features   

**Figure 2:**
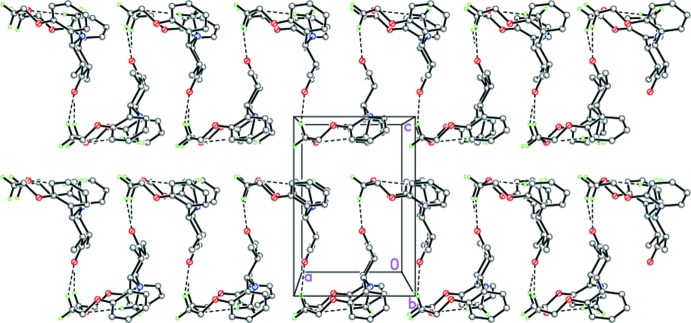
Crystal packing of (I) along the *b* axis demonstrating the zigzag hydrogen-bonded chains along [100]. Dashed lines indicate the intra- and inter­molecular C—H⋯O hydrogen bonds.

In the crystal, mol­ecules of (I) form zigzag chains along [100] *via* weak C—H⋯O inter­actions (Table 1 ▸, Figs. 2 ▸ and 3 ▸). π–π stacking is observed in the crystal, the distance between parallel benzene rings is 3.446 (3) Å and the shortest inter­molecular C5⋯C7^i^ distance is 3.495 (2) Å [symmetry code: (i) 1 − *x*, 1 − *y*, −*z*].

## Synthesis and crystallization   

Methyl­ammonium acetate (3.85 g, 50 mmol) was added to a solution of 1,5-bis­(2-formyl­phen­oxy)-3-oxa­pentane (3.14 g, 10.0 mmol) and di­ethyl­ketone (1.41 g, 10.0 mmol) in ethanol/acetic acid (40 mL/1 mL) mixture. The reaction mixture was stirred at 293 K for three days (monitored by TLC until the disappearance of the starting heterocyclic ketone spot). At the end of the reaction, the formed precipitate was filtered off, washed with ethanol and recrystallized from ethanol solution to give 2.1 g of crystals of (I) (yield 32% m.p. 485–487 K). IR (KBr), *ν*/cm^−1^: 1702. ^1^H NMR (CDCl_3_, 400 MHz, 300 K): *δ* = 0.89 (*d*, 6H, CH_3_, *J* = 6.7 Hz), 1.86 (*c*, 3H, NCH_3_), 3.19 (*d*, 2H, H1, H21, *J* = 10.8 Hz), 3.76–4.16 (*m*, 10H, H_ether_ and H22, H24), 6.78–7.26 (*m*, 8H, H_arom_). Analysis calculated for C_24_H_29_NO_4_: C, 67.72; H, 7.31; N, 5.64. Found: C, 67.54; H, 7.42; N, 5.41.

## Refinement   

Crystal data, data collection and structure refinement details are summarized in Table 2 ▸. All hydrogen atoms were placed in calculated positions with C—H = 0.95 Å (aryl-H), 0.96 Å (methyl-H), 0.98 Å (methyl­ene-H) or 1.00 Å (methine-H) and refined using a riding model with fixed isotropic displacement parameters [*U*_iso_(H) = 1.5*U*_eq_(C) for the methyl groups and 1.2*U*_eq_(C) for all other atoms].

## Supplementary Material

Crystal structure: contains datablock(s) global, I. DOI: 10.1107/S2056989016020508/xu5896sup1.cif

Structure factors: contains datablock(s) I. DOI: 10.1107/S2056989016020508/xu5896Isup2.hkl

CCDC reference: 1524447

Additional supporting information:  crystallographic information; 3D view; checkCIF report

## Figures and Tables

**Figure 3:**
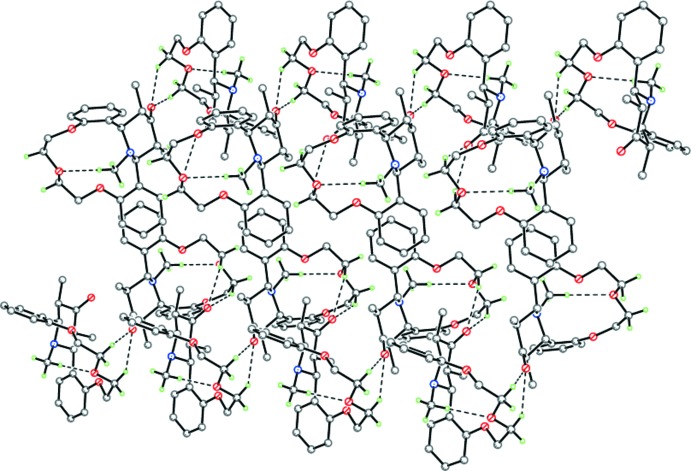
A portion of the crystal packing of (I) indicating the inter­molecular π–π stacking inter­actions. Dashed lines indicate the intra- and inter­molecular C—H⋯O hydrogen bonds.

**Table 1:** Hydrogen-bond geometry (Å, °)

*D*—H⋯*A*	*D*—H	H⋯*A*	*D*⋯*A*	*D*—H⋯*A*
C10—H10*B*⋯O12^i^	0.99	2.58	3.449 (2)	147
C16—H16*B*⋯O11	0.96	2.57	3.530 (3)	180

Symmetry code: (i) 
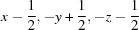
.

**Table 2:** Experimental details

Crystal data	
Chemical formula	C_24_H_29_NO_4_
*M*_r_	395.48
Crystal system, space group	Orthorhombic, *P* *n* *m* *a*
Temperature (K)	120
*a*, *b*, *c* (Å)	8.1468 (5), 20.3402 (11), 12.0155 (7)
*V* (Å^3^)	1991.1 (2)
*Z*	4
Radiation type	Mo *K*α
μ (mm^−1^)	0.09
Crystal size (mm)	0.20 × 0.15 × 0.15

Data collection	
Diffractometer	Bruker APEXII CCD
Absorption correction	Multi-scan (*SADABS*; Bruker, 2003 ▸)
*T*_min_, *T*_max_	0.970, 0.980
No. of measured, independent and observed [*I* > 2σ(*I*)] reflections	25079, 3128, 2469
*R*_int_	0.065
(sin θ/λ)_max_ (Å^−1^)	0.715

Refinement	
*R*[*F* ^2^ > 2σ(*F* ^2^)], *wR*(*F* ^2^), *S*	0.056, 0.130, 1.02
No. of reflections	3128
No. of parameters	140
H-atom treatment	H-atom parameters constrained
Δρ_max_, Δρ_min_ (e Å^−3^)	0.34, −0.23

Computer programs: *APEX2* (Bruker, 2001 ▸) and *SAINT* (Bruker, 2005 ▸), *SHELXT* (Sheldrick, 2015*a* ▸), *SHELXL2015* (Sheldrick, 2015*b* ▸) and *SHELXTL* (Sheldrick, 2008 ▸).

## supplementary crystallographic information

### Crystal data

**Table:**

C_24_H_29_NO_4_	*D*_x_ = 1.319 Mg m^−^^3^
*M**_r_* = 395.48	Mo *K*α radiation, λ = 0.71073 Å
Orthorhombic, *P**n**m**a*	Cell parameters from 4090 reflections
*a* = 8.1468 (5) Å	θ = 3.0–29.2°
*b* = 20.3402 (11) Å	µ = 0.09 mm^−^^1^
*c* = 12.0155 (7) Å	*T* = 120 K
*V* = 1991.1 (2) Å^3^	Prism, colourless
*Z* = 4	0.20 × 0.15 × 0.15 mm
*F*(000) = 848	

### Data collection

**Table:**

Bruker APEXII CCD diffractometer	2469 reflections with *I* > 2σ(*I*)
Radiation source: fine-focus sealed tube	*R*_int_ = 0.065
φ and ω scans	θ_max_ = 30.6°, θ_min_ = 2.0°
Absorption correction: multi-scan (*SADABS*; Bruker, 2003)	*h* = −11→11
*T*_min_ = 0.970, *T*_max_ = 0.980	*k* = −29→29
25079 measured reflections	*l* = −17→16
3128 independent reflections	

### Refinement

**Table:**

Refinement on *F*^2^	Primary atom site location: difference Fourier map
Least-squares matrix: full	Secondary atom site location: difference Fourier map
*R*[*F*^2^ > 2σ(*F*^2^)] = 0.056	Hydrogen site location: mixed
*wR*(*F*^2^) = 0.130	H-atom parameters constrained
*S* = 1.02	*w* = 1/[σ^2^(*F*_o_^2^) + (0.046*P*)^2^ + 1.66*P*] where *P* = (*F*_o_^2^ + 2*F*_c_^2^)/3
3128 reflections	(Δ/σ)_max_ < 0.001
140 parameters	Δρ_max_ = 0.34 e Å^−^^3^
0 restraints	Δρ_min_ = −0.23 e Å^−^^3^

### Special details

**Table:**

Geometry. All esds (except the esd in the dihedral angle between two l.s. planes) are estimated using the full covariance matrix. The cell esds are taken into account individually in the estimation of esds in distances, angles and torsion angles; correlations between esds in cell parameters are only used when they are defined by crystal symmetry. An approximate (isotropic) treatment of cell esds is used for estimating esds involving l.s. planes.

### Fractional atomic coordinates and isotropic or equivalent isotropic displacement parameters (Å^2^)

**Table:**

	*x*	*y*	*z*	*U*_iso_*/*U*_eq_	
C1	0.58338 (18)	0.30912 (7)	−0.07447 (12)	0.0151 (3)	
H1	0.4639	0.3029	−0.0903	0.018*	
C2	0.60218 (18)	0.37109 (7)	−0.00494 (12)	0.0156 (3)	
C3	0.75023 (19)	0.40480 (7)	0.00526 (13)	0.0190 (3)	
H3	0.8432	0.3900	−0.0354	0.023*	
C4	0.7652 (2)	0.45976 (7)	0.07366 (14)	0.0223 (3)	
H4	0.8670	0.4822	0.0793	0.027*	
C5	0.6301 (2)	0.48134 (7)	0.13342 (13)	0.0223 (3)	
H5	0.6398	0.5188	0.1803	0.027*	
C6	0.4808 (2)	0.44885 (7)	0.12557 (13)	0.0195 (3)	
H6	0.3888	0.4637	0.1672	0.023*	
C7	0.46690 (18)	0.39412 (7)	0.05590 (12)	0.0162 (3)	
O8	0.32384 (13)	0.35995 (5)	0.04244 (9)	0.0194 (2)	
C9	0.19912 (19)	0.36553 (8)	0.12588 (13)	0.0201 (3)	
H9A	0.1387	0.4074	0.1172	0.024*	
H9B	0.2488	0.3645	0.2011	0.024*	
C10	0.08483 (19)	0.30816 (7)	0.11056 (13)	0.0197 (3)	
H10A	−0.0119	0.3129	0.1597	0.024*	
H10B	0.0462	0.3062	0.0325	0.024*	
O11	0.17272 (19)	0.2500	0.13785 (13)	0.0192 (3)	
C12	0.6367 (3)	0.2500	−0.24937 (18)	0.0157 (4)	
O12	0.5743 (2)	0.2500	−0.34102 (13)	0.0219 (3)	
C13	0.67442 (18)	0.31314 (7)	−0.18726 (12)	0.0160 (3)	
H13	0.7951	0.3147	−0.1722	0.019*	
N14	0.6428 (2)	0.2500	−0.01435 (14)	0.0148 (3)	
C15	0.6282 (2)	0.37347 (7)	−0.25565 (13)	0.0192 (3)	
H15A	0.6833	0.3715	−0.3281	0.029*	
H15B	0.6626	0.4133	−0.2161	0.029*	
H15C	0.5091	0.3744	−0.2667	0.029*	
C16	0.6033 (3)	0.2500	0.10474 (17)	0.0186 (4)	
H16A	0.6489	0.2885	0.1390	0.028*	
H16B	0.4864	0.2500	0.1141	0.028*	

### Atomic displacement parameters (Å^2^)

**Table:**

	*U*^11^	*U*^22^	*U*^33^	*U*^12^	*U*^13^	*U*^23^
C1	0.0192 (7)	0.0111 (6)	0.0151 (6)	0.0006 (5)	0.0007 (5)	−0.0001 (5)
C2	0.0197 (7)	0.0118 (6)	0.0154 (6)	0.0002 (5)	−0.0012 (5)	0.0008 (5)
C3	0.0200 (7)	0.0152 (6)	0.0217 (7)	0.0009 (5)	−0.0016 (6)	0.0010 (5)
C4	0.0240 (8)	0.0169 (7)	0.0259 (8)	−0.0028 (6)	−0.0061 (6)	−0.0005 (6)
C5	0.0317 (8)	0.0133 (6)	0.0219 (7)	0.0005 (6)	−0.0075 (6)	−0.0042 (6)
C6	0.0248 (7)	0.0161 (7)	0.0177 (7)	0.0031 (6)	−0.0015 (6)	−0.0026 (5)
C7	0.0202 (7)	0.0124 (6)	0.0158 (7)	0.0003 (5)	−0.0018 (5)	0.0004 (5)
O8	0.0198 (5)	0.0201 (5)	0.0183 (5)	−0.0026 (4)	0.0035 (4)	−0.0054 (4)
C9	0.0208 (7)	0.0203 (7)	0.0193 (7)	0.0025 (6)	0.0038 (6)	−0.0033 (6)
C10	0.0176 (7)	0.0217 (7)	0.0199 (7)	0.0033 (6)	0.0015 (6)	−0.0008 (6)
O11	0.0177 (7)	0.0183 (7)	0.0216 (8)	0.000	−0.0020 (6)	0.000
C12	0.0152 (9)	0.0157 (9)	0.0162 (9)	0.000	0.0047 (7)	0.000
O12	0.0306 (9)	0.0190 (7)	0.0160 (7)	0.000	−0.0006 (6)	0.000
C13	0.0161 (6)	0.0149 (6)	0.0169 (7)	−0.0019 (5)	0.0014 (5)	−0.0001 (5)
N14	0.0193 (8)	0.0104 (7)	0.0147 (8)	0.000	−0.0017 (6)	0.000
C15	0.0245 (8)	0.0144 (6)	0.0187 (7)	−0.0008 (6)	−0.0002 (6)	0.0028 (5)
C16	0.0271 (11)	0.0154 (9)	0.0134 (9)	0.000	−0.0003 (8)	0.000

### Geometric parameters (Å, º)

**Table:**

C1—N14	1.4840 (17)	C9—H9B	0.9900
C1—C2	1.5197 (19)	C10—O11	1.4211 (17)
C1—C13	1.547 (2)	C10—H10A	0.9900
C1—H1	1.0000	C10—H10B	0.9900
C2—C3	1.393 (2)	O11—C10^i^	1.4211 (17)
C2—C7	1.403 (2)	C12—O12	1.213 (3)
C3—C4	1.393 (2)	C12—C13^i^	1.5168 (18)
C3—H3	0.9500	C12—C13	1.5168 (18)
C4—C5	1.385 (2)	C13—C15	1.524 (2)
C4—H4	0.9500	C13—H13	1.0000
C5—C6	1.388 (2)	N14—C16	1.467 (3)
C5—H5	0.9500	N14—C1^i^	1.4840 (17)
C6—C7	1.397 (2)	C15—H15A	0.9800
C6—H6	0.9500	C15—H15B	0.9800
C7—O8	1.3666 (18)	C15—H15C	0.9800
O8—C9	1.4319 (18)	C16—H16A	0.9595
C9—C10	1.504 (2)	C16—H16B	0.9593
C9—H9A	0.9900		
			
N14—C1—C2	111.82 (12)	H9A—C9—H9B	108.6
N14—C1—C13	108.23 (12)	O11—C10—C9	107.80 (13)
C2—C1—C13	112.92 (12)	O11—C10—H10A	110.1
N14—C1—H1	107.9	C9—C10—H10A	110.1
C2—C1—H1	107.9	O11—C10—H10B	110.1
C13—C1—H1	107.9	C9—C10—H10B	110.1
C3—C2—C7	118.03 (13)	H10A—C10—H10B	108.5
C3—C2—C1	122.95 (13)	C10—O11—C10^i^	112.69 (16)
C7—C2—C1	118.96 (13)	O12—C12—C13^i^	122.11 (9)
C2—C3—C4	121.51 (15)	O12—C12—C13	122.11 (9)
C2—C3—H3	119.2	C13^i^—C12—C13	115.71 (18)
C4—C3—H3	119.2	C12—C13—C15	111.50 (13)
C5—C4—C3	119.40 (15)	C12—C13—C1	106.80 (12)
C5—C4—H4	120.3	C15—C13—C1	113.36 (12)
C3—C4—H4	120.3	C12—C13—H13	108.3
C4—C5—C6	120.68 (14)	C15—C13—H13	108.3
C4—C5—H5	119.7	C1—C13—H13	108.3
C6—C5—H5	119.7	C16—N14—C1^i^	113.80 (10)
C5—C6—C7	119.40 (14)	C16—N14—C1	113.80 (10)
C5—C6—H6	120.3	C1^i^—N14—C1	108.27 (15)
C7—C6—H6	120.3	C13—C15—H15A	109.5
O8—C7—C6	123.03 (14)	C13—C15—H15B	109.5
O8—C7—C2	116.00 (12)	H15A—C15—H15B	109.5
C6—C7—C2	120.97 (14)	C13—C15—H15C	109.5
C7—O8—C9	118.82 (11)	H15A—C15—H15C	109.5
O8—C9—C10	106.99 (12)	H15B—C15—H15C	109.5
O8—C9—H9A	110.3	N14—C16—H16A	109.5
C10—C9—H9A	110.3	N14—C16—H16B	109.4
O8—C9—H9B	110.3	H16A—C16—H16B	109.5
C10—C9—H9B	110.3		
			
N14—C1—C2—C3	80.51 (18)	C2—C7—O8—C9	159.44 (13)
C13—C1—C2—C3	−41.84 (19)	C7—O8—C9—C10	−161.82 (12)
N14—C1—C2—C7	−96.59 (16)	O8—C9—C10—O11	67.31 (16)
C13—C1—C2—C7	141.06 (13)	C9—C10—O11—C10^i^	−171.15 (10)
C7—C2—C3—C4	0.1 (2)	O12—C12—C13—C15	−1.4 (2)
C1—C2—C3—C4	−177.05 (14)	C13^i^—C12—C13—C15	−178.58 (11)
C2—C3—C4—C5	0.3 (2)	O12—C12—C13—C1	122.9 (2)
C3—C4—C5—C6	−0.1 (2)	C13^i^—C12—C13—C1	−54.2 (2)
C4—C5—C6—C7	−0.5 (2)	N14—C1—C13—C12	58.80 (16)
C5—C6—C7—O8	−179.15 (14)	C2—C1—C13—C12	−176.87 (13)
C5—C6—C7—C2	0.9 (2)	N14—C1—C13—C15	−178.02 (12)
C3—C2—C7—O8	179.34 (13)	C2—C1—C13—C15	−53.68 (16)
C1—C2—C7—O8	−3.41 (19)	C2—C1—N14—C16	38.57 (19)
C3—C2—C7—C6	−0.7 (2)	C13—C1—N14—C16	163.56 (14)
C1—C2—C7—C6	176.59 (13)	C2—C1—N14—C1^i^	166.15 (9)
C6—C7—O8—C9	−20.6 (2)	C13—C1—N14—C1^i^	−68.85 (18)

Symmetry code: (i) *x*, −*y*+1/2, *z*.

### Hydrogen-bond geometry (Å, º)

**Table:**

*D*—H···*A*	*D*—H	H···*A*	*D*···*A*	*D*—H···*A*
C10—H10*B*···O12^ii^	0.99	2.58	3.449 (2)	147
C16—H16*B*···O11	0.96	2.57	3.530 (3)	180

Symmetry code: (ii) *x*−1/2, −*y*+1/2, −*z*−1/2.

## References

[bb1] An, H., Wang, T., Mohan, V., Griffey, R. H. & Cook, P. D. (1998). *Tetrahedron*, **54**, 3999–4012.

[bb2] Anh, L. T., Hieu, T. H., Soldatenkov, A. T., Kolyadina, N. M. & Khrustalev, V. N. (2012*b*). *Acta Cryst.* E**68**, o1588–o1589. 10.1107/S1600536812018867 PMC3379201 22719399

[bb3] Anh, L. T., Hieu, T. H., Soldatenkov, A. T., Kolyadina, N. M. & Khrustalev, V. N. (2012*c*). *Acta Cryst.* E**68**, o2165–o2166. 10.1107/S1600536812027274 PMC3393972 22798837

[bb4] Anh, L. T., Hieu, T. H., Soldatenkov, A. T., Soldatova, S. A. & Khrustalev, V. N. (2012*a*). *Acta Cryst.* E**68**, o1386–o1387. 10.1107/S1600536812015206 PMC3344514 22590276

[bb5] Anh, L. T., Levov, A. N., Soldatenkov, A. T., Gruzdev, R. D. & Hieu, T. H. (2008). *Russ. J. Org. Chem.* **44**, 463–465.

[bb6] Artiemenko, A. G., Kovdienko, N. A., Kuz’min, V. E., Kamalov, G. L., Lozitskaya, R. N., Fedchuk, A. S., Lozitsky, V. P., Dyachenko, N. S. & Nosach, L. N. (2002). *Exp. Oncol.* **24**, 123–127.

[bb7] Bradshaw, J. S. & Izatt, R. M. (1997). *Acc. Chem. Res.* **30**, 338–345.

[bb8] Bruker (2001). *SAINT*. Bruker AXS Inc., Madison, Wisconsin, USA.

[bb9] Bruker (2003). *SADABS*. Bruker AXS Inc., Madison, Wisconsin, USA.

[bb10] Bruker (2005). *APEX2*. Bruker AXS Inc., Madison, Wisconsin, USA.

[bb11] Costero, A. M., Bañuls, M. J., Aurell, M. J., Ochando, L. E. & Doménech, A. J. (2005). *Tetrahedron*, **61**, 10309–10320.

[bb12] Gökel, G. W. & Murillo, O. (1996). *Acc. Chem. Res.* **29**, 425–432.

[bb13] Hieu, T. H., Anh, L. T., Soldatenkov, A. T., Golovtsov, N. I. & Soldatova, S. A. (2011). *Chem. Heterocyc. Compd.* **47**, 1307–1308.

[bb14] Hieu, T. H., Anh, L. T., Soldatenkov, A. T., Kolyadina, N. M. & Khrustalev, V. N. (2012*a*). *Acta Cryst.* E**68**, o2431–o2432. 10.1107/S1600536812030644 PMC3414347 22904880

[bb15] Hieu, T. H., Anh, L. T., Soldatenkov, A. T., Kurilkin, V. V. & Khrustalev, V. N. (2012*b*). *Acta Cryst.* E**68**, o2848–o2849. 10.1107/S1600536812037051 PMC3470208 23125652

[bb16] Le, A. T. & Soldatenkov, A. T. (2016). *Chem. Heterocycl. Compd*, **52**, 152–154.

[bb17] Le, T. A., Truong, H. H., Nguyen, P. T. T., Pham, H. T., Kotsuba, V. E., Soldatenkov, A. T., Khrustalev, V. N. & Wodajo, A. T. (2014). *Macroheterocycles*, **7**, 386–390.

[bb18] Le, A. T., Truong, H. H., Thi, T. P. N., Thi, N. D., To, H. T., Thi, H. P. & Soldatenkov, A. T. (2015). *Mendeleev Commun.* **25**, 224–225.

[bb19] Levov, A. N., Anh, L. T., Komarova, A. I., Strokina, V. M., Soldatenkov, A. T. & Khrustalev, V. N. (2008). *Russ. J. Org. Chem.* **44**, 457–462.

[bb20] Levov, A. N., Strokina, V. M., Anh, L. T., Komarova, A. I., Soldatenkov, A. T. & Khrustalev, V. N. (2006). *Mendeleev Commun.* **16**, 35–36.

[bb21] Natali, M. & Giordani, S. (2012). *Chem. Soc. Rev.* **41**, 4010–4029. 10.1039/c2cs35015g 22426200

[bb22] Pedersen, C. J. (1988). *Angew. Chem.* **100**, 1053–1059.

[bb23] Sheldrick, G. M. (2008). *Acta Cryst.* A**64**, 112–122. 10.1107/S0108767307043930 18156677

[bb24] Sheldrick, G. M. (2015*a*). *Acta Cryst.* A**71**, 3–8.

[bb25] Sheldrick, G. M. (2015*b*). *Acta Cryst.* C**71**, 3–8.

[bb26] Tran, T. T. V., Anh, L. T., Nguyen, H. H., Truong, H. H. & Soldatenkov, A. T. (2016). *Acta Cryst.* E**72**, 663–666. 10.1107/S2056989016005752 PMC4908512 27308014

